# Dissipation behavior and dietary risk assessment of pesticide residues during the winemaking of *Fetească neagră* and C*abernetSauvignon*

**DOI:** 10.1016/j.fochx.2026.103930

**Published:** 2026-05-10

**Authors:** Georgiana-Diana Gabur, Valeriu V. Cotea, Daniela Fighir, Carmen Teodosiu, Iulian Gabur

**Affiliations:** a “Ion Ionescu de la Brad” Iasi University of Life Sciences, Faculty of Horticulture, 3 Mihail Sadoveanu Alley, 700490, Iasi, Romania; b “Gheorghe Asachi” Technical University of Iasi, Faculty of Chemical, Engineering and Environmental Protection, 73 D. Mangeron Street, 700050 Iasi, Romania; c “Ion Ionescu de la Brad” Iasi University of Life Sciences, Faculty of Agriculture, 3 Mihail Sadoveanu Alley, 700490 Iasi, Romania

**Keywords:** Pesticide residues, Red winemaking, LC-MS/MS, QuEChERS, Processing factor, Risk assessment

## Abstract

Pesticide residue persistence in viticulture poses significant challenges for food safety and oenological integrity. This study investigated the dissipation kinetics and fate of seven pesticides during the winemaking of two red varieties, *Fetească neagră* and *Cabernet Sauvignon*. Residues were tracked across six processing stages, from fresh grapes to filtered wine. Pesticide residues were extracted following the QuEChERS protocol and subsequently quantified using LC-MS/MS. The study demonstrates a significant change of pesticide residues throughout the entire winemaking process, with cumulative reduction rates up to 89.70% for *Fetească neagră* and to 75.74% for *Cabernet Sauvignon*, confirming that wine processing acts as a highly effective mitigation stage for chemical residues. Physicochemical sequestration during red wine processing serves as a critical safety barrier, maintaining toxicological risks (HI < 1) within safe limits. Our findings provide a predictive framework for residue behavior, demonstrating that pesticide physicochemical properties and vinification techniques interact to effectively safeguard consumer health.

## Highlights

• Pesticide residues significantly decreased throughout all winemaking stages.
• LC-MS/MS tracked seven pesticides across six stages of red winemaking.
• Varietal-specific matrix effects significantly influence residue dissipation.
• Iprovalicarb and myclobutanil reached the highest reduction rates (up to 89%).
• Risk assessment (HQ/HI) confirmed no health risk for wine consumers.

## Graphical abstract

**Figure:**
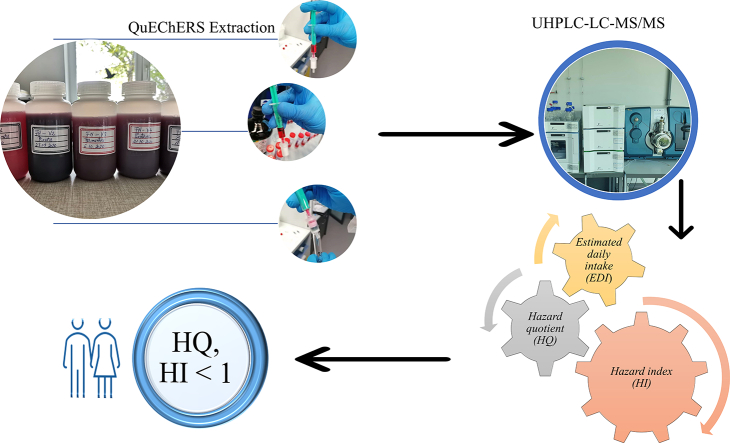

## 1 Introduction

Food safety has emerged as a critical global concern, with increasing focus shifting from basic nutritional adequacy to the physicochemical quality, diversity, and toxicological integrity of food matrices. Within this framework, viticulture faces a significant challenge, as pesticide residues represent a major public health concern due to their potential for inappropriate application and environmental persistence (Yang et al., 2023). While pesticides are pivotal in mitigating crop losses, their tendency to accumulate in grapes and subsequently transfer into the final product necessitates rigorous monitoring. Consequently, understanding the fate and redistribution of these chemical residues throughout the winemaking process is essential to ensure consumer safety and regulatory compliance.

Dietary intake constitutes the primary pathway for human exposure to pesticides and related chemical contaminants, underscoring the imperative for rigorous monitoring of residue levels in high-consumption commodities such as grapes and wine. Conventional food safety assessments have traditionally focused on individual chemical entities; however, real-world dietary exposure typically involves complex multi-residue mixtures (European Food Safety Authority (EFSA), 2022). Consequently, international regulatory bodies emphasize the need for integrated safety metrics. To address this, toxicological indices such as the Estimated Daily Intake (EDI), the Hazard Quotient (HQ) for individual compounds, and Hazard Index (HI) for cumulative effects have become essential tools in quantifying the non-carcinogenic health risks posed by concurrent exposure to multiple residues in the human diet.

Among horticultural crops, the grapevine *(Vitis vinifera L.)* represents one of the most historically significant and economically vital species globally, with a cultivation footprint of approximately 7.5 million hectares and an annual yield exceeding 90 million tons (Dahlawi et al., 2021). Approximately 75% of the global grape harvest is channeled into winemaking, underscoring the profound economic and social impact of the viti-vinicultural sector (Zhu et al., 2015). Beyond oenological use, grapes are extensively consumed as fresh table fruit or processed into secondary commodities such as juices, raisins, and vinegars. These processing pathways not only extend shelf life but also enhance the economic value of the grape-derived matrices, necessitating a thorough understanding of their chemical safety profiles.

Nevertheless, grapevine cultivation is highly vulnerable to a wide range of fungal diseases, including downy mildew (*Plasmopara viticola*), *Eutypa* dieback (*Eutypa lata*), black rot (*Guignardia bidwellii*), *Botrytis* bunch rot (*Botrytis cinerea*), *Phomopsis* cane and leaf spot (*Phomopsis viticola*), powdery mildew (*Uncinula necator*), and sour rot, which can be caused by several fungi, such as A*spergillus niger, Alternaria tenuis, Botrytis cinerea, Cladosporium herbarum, Rhizopus arrhizus*, and *Penicillium* spp. (Sonker et al., 2016).

To ensure stable yields and meet commercial quality standards, viticulture frequently relies on the intensive application of fungicides and other plant protection products. Trace amounts of these xenobiotics, termed residues, can persist within the grape matrix and may be transferred into processed derivatives. Consequently, improper or excessive application can lead to pesticide accumulation, potentially compromising toxicological safety and creating barriers to international trade (Shabeer et al., 2015).

The transformation of grapes into wine significantly modulates residue levels. The Processing Factor (PF) serves as a critical analytical tool to quantify these fluctuations, defined as the ratio of residue concentration in the processed product to that in the raw agricultural commodity (FAO, 2016). A PF < 1 denotes a reduction in residue levels, while a PF > 1 indicates concentration effects (Han et al., 2014). This factor is governed by the physicochemical properties of the pesticides, such as the octanol-water partition coefficient (K_ow_) and water solubility, alongside oenological practices (e.g., maceration, fermentation, and clarification) and the biological characteristics of the grape cultivar (Quan et al., 2020).

A comprehensive understanding of the fate of pesticide residues throughout the winemaking process is indispensable for accurate dietary risk assessments. Reliable data on Processing Factors (PFs) facilitate more realistic estimations of consumer exposure and provide a scientific basis for optimizing pre-harvest intervals (PHIs). These insights are crucial for ensuring that chemical residues in the final oenological products remain strictly within regulatory thresholds, thereby safeguarding both public health and market access.

In the European Union, maximum residue limits (MRLs) are established for pesticides in raw grapes intended for winemaking; however, no specific MRLs exist for the final wine product. Consequently, it is generally assumed that the MRLs for raw grapes also apply to processed products (European Commission, 2024; Romero-González et al., 2011). Several technological steps in winemaking, including pressing, fermentation, and filtration, can substantially influence pesticide residue levels, often reducing residues in the finished wine (Alister et al., 2014; Kittelmann et al., 2022; Martín-García et al., 2024). Nonetheless, some pesticides, such as pyrimethanil, have been shown to persist at similar concentrations in both grapes and the final wine product (Martins et al., 2011).

The determination of pesticide residues in grapes and wine has greatly benefited from advances in analytical technologies. Liquid chromatography coupled with tandem mass spectrometry (LC-MS/MS), combined with the Quick, Easy, Cheap, Effective, Rugged, and Safe (QuEChERS) method, has proven highly effective, offering excellent sensitivity and selectivity even in complex matrices containing polyphenols, sugars, and organic acids (Christodoulou et al., 2015; Grimalt & Dehouck, 2016). Several studies have evaluated the transfer of pesticide residues from grapes to processed products such as raisins and wine, showing that winemaking generally reduced residue level through processing-induced removal and degradation (Shabeer et al., 2024). Earlier work indicated that many conventional fungicides exhibit processing factors below 1.00 when grapes are transformed into wine, reflecting significant losses during destemming, pressing, fermentation and clarification (Cabras & Angioni, 2000). More recent studies on raisins and other dried grape products further demonstrated that thermal and air-drying steps can either concentrate residues or promote their degradation, depending on the compound's physicochemical properties (Dumitriu Gabur et al., 2022; Shabeer et al., 2024). However, the behavior and fate of newer fungicides used in modern viticulture, especially under varying climatic and agronomic conditions, remain poorly characterized. This variability emphasizes the necessity of establishing locally adapted processing factors and transfer rates.

Despite the extensive knowledge already available on the dissipation of pesticides during winemaking, there is a critical lack of comparative data on how varietal-specific matrix effects, particularly in regional versus international cultivars, influence the fate of modern pesticides such as oxathiapiprolin and chlorantraniliprole. To address this, the present study was designed with the following objectives: (i) quantifying the dissipation kinetics and fate of seven priority pesticides (acetamiprid, chlorantraniliprole, deltamethrin, iprovalicarb, myclobutanil, tebuconazole and oxathiapiprolin) throughout six distinct stages of red winemaking; (ii) evaluating the influence of the grape matrix by comparing the Romanian indigenous variety *Fetească neagră* with the international variety *Cabernet Sauvignon*; (iii) assessing the potential health risks for consumers using EDI, HQ and HI indices for both gender groups. This research provides the first comprehensive dissipation framework for these specific active compounds in *Fetească neagră*, offering new insights into how varietal characteristics and oenological interventions synergistically drive residue mitigation. By establishing precise processing factors (PFs), this study addresses a significant gap in the international literature concerning the chemical integrity and safety of regional red wines in relation to global food safety standards.

## 2 Materials and methods

### 2.1 Winemaking process at pilot scale and samples collection

Mature grape samples were harvested from a 12-ha experimental vineyard located in North-Eastern Romania, at the Vasile Adamachi Farm (Iași County), affiliated with Iași University of Life Sciences “Ion Ionescu de la Brad” (IULS). Between May and August 2020, the vineyard was subjected to phytosanitary treatments, with pesticides applied at the doses recommended by the manufacturers. The products used included Zorvec Zelavin (oxathiapiprolin), Systhane (myclobutanil), Melody Compact (8.5% iprovalicarb +40.6% copper oxychloride), Folicur (tebuconazole), Coragen (chlorantraniliprole), Decis Expert (deltamethrin) and Gazelle (acetamiprid). The treatments and application rates are summarized in Supplementary Table 1.

Grapes of *Fetească neagră* and *Cabernet Sauvignon* varieties were hand-harvested at technological maturity and immediately transported to the Oenology Laboratory of Iași University of Life Sciences for processing and analysis. Sampling was conducted on September 21, 2020, for *Fetească neagră* and October 15, 2020, for *Cabernet Sauvignon*, ensuring that the fruit was processed under controlled conditions to maintain the integrity of the chemical residues.

**Fig. 1:**
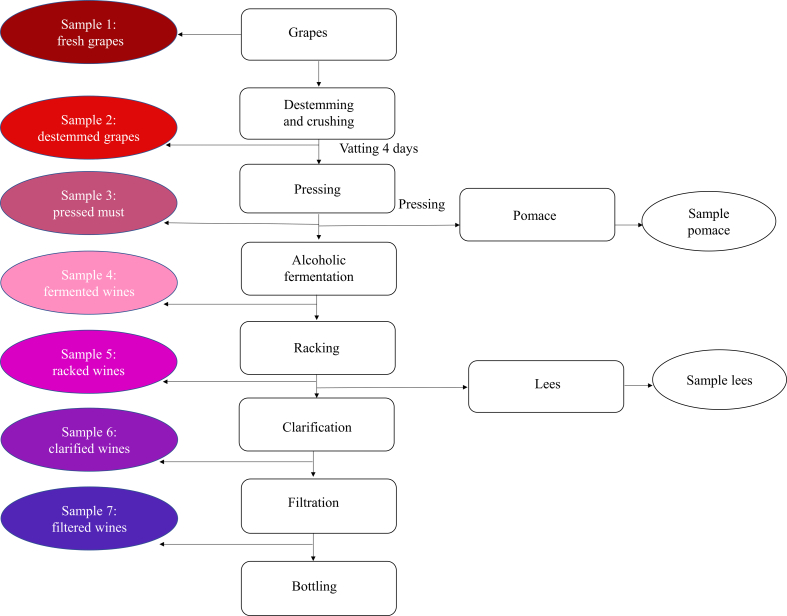
Technological flow of the winemaking process and sampling points (Samples 1-7). Samples were collected at each key stage: (1) fresh grapes, (2) destemmed and crushed grapes, (3) pressed must, (4) fermented wines, (5) racked wines, (6) clarified wines, and (7) filtered wines

The winemaking process followed the technological flow illustrated in Fig. 1 and was identical for both grape varieties. Samples were collected at key stages of winemaking (Samples 1–7) for subsequent analysis. Fresh grapes (Sample 1) were destemmed and crushed to obtain the grape must, consisting of a liquid fraction (must) and a solid fraction (pomace, collected separately as the pomace sample). During this stage, samples of destemmed and crushed grapes were collected (Sample 2). The crushed grapes were subjected to vatting for four days at temperatures below 10 °C to promote the extraction of phenolic and aromatic compounds. After maceration, the must was pressed, resulting in pressed must (Sample 3) and pomace. The must was then transferred into stainless steel fermentation tanks and inoculated with the active dry yeast *Saccharomyces cerevisiae* (Xr Grand Rouge, Lamothe-Abiet), together with enzymatic preparations OptiEsters™ (30 g/hL) and OptiThiols™ (as recommended by the manufacturer). Alcoholic fermentation was conducted under controlled temperature conditions (20 ± 2 °C) and samples of fermented wines were collected (Sample 4). After malolactic fermentation, the wines were racked to separate the lees (collected as the lees sample), yielding the racked wines (Sample 5) and then sulfur dioxide (SO₂) was added. The wines were subsequently ageing, clarified (Sample 6), filtered (Sample 7), and finally bottled and stored in a temperature-controlled dark cellar until analysis.

### 2.2 Sample preparation and analytical measurement

#### 2.2.1 Reagents and materials

Pesticide standards were obtained from Dr. Ehrenstorfer GmbH (Augsburg, Germany). A mixed standard solution (DRE-Q60 004725) containing 10 μg/mL each of acetamiprid, chlorantraniliprole, and iprovalicarb was used, while individual standard solutions at 10 μg/mL in acetonitrile were prepared for the remaining pesticides, also from Dr. Ehrenstorfer. Dilutions to the required concentrations were carried out in acetonitrile. The final working solution was prepared by mixing 0.25 mL of the standard solution with 10 μL of the internal standard (Triphenyl phosphate - TPP, 5 μg/mL) and 0.74 mL of ultrapure water.

Quick QuEChERS-Medium Cartridges (United Chemical Technologies (Bristol, PA, USA) contain 110 mg anhydrous magnesium sulfate (MgSO_4_) and 190 mg primary-secondary amine (PSA) as sorbent for the removal of polar matrix interferences during clean-up.

Extraction salts, including magnesium sulfate (MgSO₄), sodium chloride (NaCl), acetonitrile, 2-propanol, and acetone, were purchased from Merck KGaA (Darmstadt, Germany). Formic acid (≥95%) was obtained from Fisher Chemical, and ammonium formate from Sigma-Aldrich (St. Louis, MO, USA). Methanol was supplied by HiPerSolv, VWR Chemicals (Radnor, PA, USA). Chromatographic separations were performed using a Kinetex biphenyl column (2.6 μm, 4.6 × 150 mm; Phenomenex, Torrance, CA, USA), and ultrapure water was produced using a Milli-Q water purification system (Millipore, Bedford, MA, USA).

#### 2.2.2 QuEChERS extraction

For the QuEChERS procedure, 10 mL of each sample was transferred to a 50 mL polypropylene centrifuge tube, followed by the addition of 10 mL of acetonitrile. The mixture was vortexed for 30 s, after which a salt mixture (4 g MgSO₄ and 2 g NaCl) was added and the tube was shaken using a rotary stirrer for 1 min to enhance phase separation in the grape-must matrix. Samples were then centrifuged for 5 min at 5000 rpm. From the resulting supernatant, 1 mL was withdrawn using a syringe and passed through a quick QuEChERS cartridge, in accordance with the manufacturer's clean-up guidelines. Subsequently, 0.25 mL of the filtrate was transferred to a 2 mL autosampler vial, to which 10 μL of the internal standard (TPP, 5 μg/mL) and 0.74 mL of ultrapure water were added. This final dilution step was performed to minimize chromatographic band broadening due to solvent mismatch. The prepared extract was then injected into the LC-MS/MS system for analysis.

#### 2.2.3 Equipment and instrumental parameters

Pesticide determinations were performed using an ultra-high performance liquid chromatography, UHPLC Flexar chromatographic system (Perkin Elmer, Waltham, MA, USA) equipped with a Kinetex biphenyl column (4.6 × 150 mm, 2.6 μm particle size; Phenomenex, Torrance, CA, USA). Detection was carried out on an AB SCIEX Triple Quad 5500 mass spectrometer (Framingham, MA, USA), and data analysis was conducted using SCIEX Analyst 2.0 software. The working solutions for the mobile phases were prepared as follows: (a) Mobile phase A (MPA): 315.28 mg of ammonium formate was dissolved in 1 L of ultrapure water, followed by the addition of 1 mL formic acid. (b) Mobile phase B (MPB): 315.28 mg of ammonium formate was first dissolved in 20 mL ultrapure water, then diluted to 1 L with methanol and homogenized. The high-performance liquid chromatography (HPLC) pump was operated at a flow rate of 1 mL/min with a gradient elution program detailed in Table 1.

**Table 1:** Mobile phase gradient conditions.

Elution time (min)	Mobile phase A %	Mobile phase B %
0.70	95	5
1.00	50	50
1.50	40	60
2.50	22	78
4.00	12	88
10.00	8	92
12.00	0	100
24.00	0	100
25.00	95	5
29.50	95	5

The injection volume was finalized at 20 μL. The sample compartment temperature was maintained at 10 °C, while the column oven was kept at 33 °C. The ionization source temperature was set to 500 °C. The gas settings were as follows: curtain gas at 35 psi, collision gas at 9 psi, ion spray voltage at 3500 V, and both ion source gas 1 and gas 2 set to 60 psi.

Analyses were performed using positive electrospray ionization (ESI) in retention time-scheduled multiple reaction monitoring (MRM) mode, acquiring one transition for quantification and a second for confirmation of each analyte. The data obtained are summarized in Table 2. The area ratio of the two transitions in the sample chromatograms was within ±20% of the average area ratio observed in the standard solution chromatograms, thus ensuring reliable analyte identification. Matrix-matched calibration standards (CC1–6: 0.6–12.5 ng/mL) were prepared by spiking the pesticide mixed stock solution (MSS, 500 ng/mL) into blank wine extracts processed via QuEChERS. Final sample concentrations were calculated using Csmpl.u = Cstd.u × (10/4), accounting for extraction (10 mL) and final dilution (4-fold). LOD/LOQ were estimated from S/N ratios (3,1 and 10:1, respectively) using *n* = 5 replicates per level, based on the quantifier MRM transition (Q1/Q3), data provided in Supplementary Table 2.

**Table 2:** Physicochemical properties of pesticides.

Name of the compound	Molecular Formula	CAS	Molecular Mass	Solubility in water (mg/L)	log P	MRL wines (ng/g)
Acetamiprid	C_10_H_11_C_l_N_4_	160,430–64-8	222.67	4.25	0.80	800
Chlorantraniliprole	C_18_H_14_BrCl_2_N_5_O_2_	500,008–45-7	483.1	1.02	2.76	1000
Deltamethrin	C_22_H_19_Br_2_NO_3_	52,918–63-5	505.2	0.002	6.20	200
Iprovalicarb	C_18_H_28_N_2_O_3_	140,923–17-7	320.4	13.22	3.20	2000
Myclobutanil	C_15_H_17_ClN_4_	88,671–89-0	288.77	132	2.89	1500
Tebuconazole	C_16_H_22_ClN_3_O	107,534–96-3	307.82	32	3.70	1000
Oxathiapiprolin	C_24_H_22_F_5_N_5_O_2_S	1,003,318–67-9	539.5	0.1749	3.67	700

Solubility in water and log *P* values were obtained from PubChem (National Center for Biotechnology Information, https://pubchem.ncbi.nlm.nih.gov). The MRLs for wine (ng/g) correspond to the maximum residue levels established by the European Union under Regulation (EC) No 396/2005, unless otherwise specified.

### 2.3 Data analysis

In this study, grape samples were harvested without any prior washing to preserve the natural pesticide residue levels present on the fruit surface. Consequently, no initial washing step was performed, and pesticide residues were analyzed directly on the harvested grapes. This approach enabled an accurate assessment of pesticide residues as they exist in the vineyard, providing a realistic evaluation of pesticide levels before any post-harvest processing.

The percentage reduction of pesticides at each winemaking stage was calculated using the following eq. 1:

(1) $$\text{Reduction}\;\left(\%\right)=\left(1-\begin{array}{c} \frac{\text{Pesticide residue concentration after processing stage}}{\text{Pesticide residue concentration before processing stage}}\\ \; \end{array}\right)\;x\;100$$

The transfer rate of pesticide residues from grapes to wine throughout different stages of the winemaking process was assessed. The transfer rate (%) for each stage was calculated using the eq. 2:

(2) $$\text{Transfer rate}\;\left(\%\right)=\frac{\text{Pesticide residue concentration in the sample after processing stage}}{\text{Pesticide residue concentration in the initial grape sample}}\;x\;100$$

where pesticide residue concentrations correspond to the measured values in samples taken before and after each respective processing stage.

The processing factor (PF) of pesticide residues was calculated for each winemaking stage to evaluate changes in residue levels throughout processing. The PF was determined using the following eq. 3:

(3) $$\text{Processing factor}\;\left(\mathrm{PF}\right)=\frac{\text{Pesticide residue concentration after processing stage}}{\text{Pesticide residue concentration before processing stage}}\;$$

A PF value lower than 1 indicates a reduction of pesticide residues during processing, while a PF greater than 1 suggests concentration or persistence. These calculations were applied sequentially across all winemaking stages, from grapes to the final bottled wine.

### 2.4 Assessment of dietary exposure and health risk

The estimated daily intake of pesticide residues through wine consumption is influenced by the pesticide concentration in the wine, the amount of wine consumed, and the consumer's body weight (Dumitriu Gabur et al., 2021). In this study, dietary exposure was assessed separately for women and men, using body weights of 72 kg and 85 kg, respectively. Daily wine consumption was set at 150 mL for women and 300 mL for men, in line with recommendations from the International Organisation of Vine and Wine (OIV, 2019) and the Health Promotion and Gateway (2025).

The Estimated Daily Intake (EDI) of each pesticide, expressed in mg/kg body weight/day, was calculated according to eq. 4. This involved multiplying the average concentration of pesticide residues in wine (C, μg/L) by the daily wine consumption rate (R, L/day) and dividing the result by the average body weight (BW, kg) (Semla et al., 2018):

(4) EDI=C×R/BW

where:

EDI is the estimated daily intake (mg/kg bw/day); C is the concentration of pesticide residues in wine (μg/L); R is the daily wine consumption rate for adult drinkers (L/day), 0.15 L for women and 0.30 L for men; BW is the average body weight of the population assessed (kg), 72 kg for women and 85 kg for men.

The Hazard Quotient (HQ) was calculated (eq. 5) by dividing the Estimated Daily Intake (EDI) by the reference threshold known as the Acceptable Daily Intake (ADI), which in this study was considered equivalent to the reference dose (RfD):

(5) HQ=EDI/RfD

The Acceptable Daily Intake (ADI) values used for calculating the Hazard Quotient (HQ) were as follows: 0.025 mg/kg bw/day for acetamiprid and myclobutanil, 1.56 mg/kg bw/day for chlorantraniliprole, 0.010 mg/kg bw/day for deltamethrin, 0.015 mg/kg bw/day for iprovalicarb, 0.030 mg/kg bw/day for tebuconazole, and 0.140 mg/kg bw/day for oxathiapiprolin.

The cumulative risk from pesticide residue exposure was evaluated using the Hazard Index (HI) approach (eq. 6). An HI value greater than 1 signifies that the non-carcinogenic risk exceeds acceptable levels for the target population (Dumitriu Gabur et al., 2022).

(6) $$\mathrm{Hazard}\;\mathrm{index}\;\left(\mathrm{HI}\right)=\sum \mathrm{HQ}\;$$

### 2.5 Statistical analysis

A heatmap analysis was performed to highlight and compare the differences among the winemaking stages based on the amount of pesticide residues quantified in each sample. Prior to visualization, the dataset was standardized to ensure comparability between variables with different concentration ranges. The heatmap was constructed using hierarchical clustering algorithms applied both to samples and to pesticide compounds, allowing the identification of grouping patterns and similarity relationships. ANOVA and Tukey HSD post-hoc tests with compact letter display identified significant differences (*p* < 0.05) between stages for each compound. Statistical data processing, graphical representation, and interpretation of the results were carried out using the R statistical language (version 4.5.3), packages “tidyverse”, “rstatix”, “multcompView”, within the RStudio environment (RStudio Inc., Boston, MA, USA). The experimental data were collected in triplicate, and the experimental results are presented as the mean values and standard deviations.

## 3 Results and discussion

### 3.1 Dissipation behavior of pesticide residues during winemaking

The first step in understanding the effect of pesticides on winemaking is to evaluate how they dissipate. This section outlines the overall decline in residue levels from grapes to finished wine, focusing on the main stages at which significant reductions occur.

**Fig. 2:**
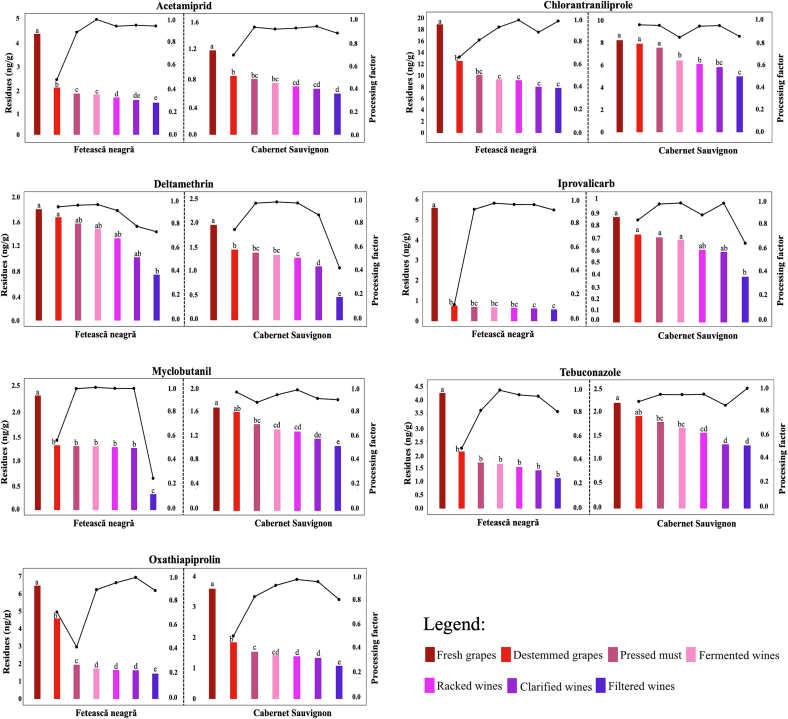
Pesticide residues and processing factors during winemaking stages for *Fetească neagră* and *Cabernet Sauvignon.* Different superscript letters indicate significant differences between winemaking stages within each compound and wine varieties (one-way ANOVA, Tukey's HSD, *p* < 0.05).

Initial concentrations were notably higher in red grape varieties (*Fetească neagră* and *Cabernet Sauvignon*), due to direct vineyard exposure and the specific canopy architecture of these varieties. As shown in Fig. 2 chlorantraniliprole and oxathiapiprolin exhibited the highest initial residues, while deltamethrin and myclobutanil remained at the lower end of the detection spectrum.

This initial persistence is influenced by both environmental degradation factors (e.g., photodegradation, temperature) and the intrinsic physicochemical properties of the compounds (Katagi, 2004).

Throughout the winemaking process, a continuous decline in pesticide level was observed, with the most significant reductions occurring during fermentation. The efficiency of pesticide residue removal was closely correlated with the water solubility and systemic nature of the compounds. High-solubility pesticides, such as myclobutanil (132 mg/L), iprovalicarb (13.22 mg/L), tebuconazole (32 mg/L) showed the highest removal rates. Conversely, lipophilic compounds with low water solubility, such as deltamethrin (0.002 mg/L) and oxathiapiprolin (0.17 mg/L) proven more persistent in the finished wine (Table 2). These findings consistent with the sequestration of hydrophobic pesticides into skins, pomace and yeast lees during alcoholic fermentation, rather than their substantial degradation by *Saccharomyces cerevisiae*, as reported in previous studies (Guo et al., 2023).

The dissipation followed a biphasic pattern: an initial rapid decline followed by a slower stabilization phase. Unlike typical environmental degradation, the pronounced early reduction in this study was driven by the physical separation of pesticide-laden solids (pomace) rather than chemical degradation. This behavior is closely linked to the octanol-water partition coefficients (log *P* values; Table 2), which dictate their distribution of residues between solid skins and liquid must. Post-fermentation, the dissipation rate decreased, likely due to the stabilizing effect of increased acidity and ethanol concentrations, on the remaining residual matrix, a trend consistent with the finding by Hou et al. (2020) for various fungicides.

Finally, while the biphasic model remained consistent, the absolute transfer rates are modulated by a complex interplay of agronomic and environmental factors and the growth dilution effect (Cui et al., 2024).

### 3.2 Transfer rate (TR) and processing factors (PF) during winemaking

The transfer rate (TR) quantifies pesticide residues movement from fresh grapes through destemmed grapes, pressed must, racked wines, clarified wines, and filtered wines, while processing factors (PF = defined as TR/100) measure concentrations changes at each stage. The results, summarized in Table 3 and Fig. 2, demonstrate that winemaking acts as a natural purification system, consistently yielding PF values below 1.00. The statistical analysis, based on ANOVA followed by Tukey's HSD test (*p* < 0.05), indicates that pesticides levels changed significantly across the winemaking stages, confirming that residue dissipation occurred progressively during wine processing. The systematic shift in statistical significance across these stages (from group ‘a’ to ‘f’ in Table 3) indicates that winemaking is acting as a multi-stage extraction process governed by distinct physicochemical drivers. The greatest pesticides reductions were observed during early stages, particularly after destemming and pressing, whereas later stages showed smaller but still statistically significant decreases. These stage-dependent differences reflect the combined effects of solid removal, fermentation, clarification and filtration on pesticides residue and overall wine safety.

**Table 3:** Reduction (%) and transfer rate (%) of the pesticide in different processing stages. Different superscript letters indicate significant differences (ANOVA and Tukey's HSD, *p* < 0.05).

***Fetească neagră***												
	Destemmed grapes		Pressed must		Fermentation		Racked wines		Clarified wines		Filtered wines	
	Red (%)	Transf (%)	Red (%)	Transf (%)	Red (%)	Transf (%)	Red (%)	Transf (%)	Red (%)	Transf (%)	Red (%)	Transf (%)
ACE	53.1^a^	46.9^A^	12.8^b^	40.9^B^	1.9^c^	40.1^C^	7.8^d^	36.9^D^	6.9^e^	34.4^E^	7.6^f^	31.8^F^
CHL	33.8^a^	66.2^A^	19.0^b^	53.6^B^	7.8^c^	49.4^C^	1.6^d^	48.6^D^	12.3^e^	42.6^E^	2.6^f^	41.5^F^
DEL	7.4^a^	92.6^A^	5.9^b^	87.1^B^	5.5^c^	82.3^C^	10.2^d^	73.9^D^	23.2^e^	56.8^E^	27.5^f^	41.2^F^
IPR	86.3^a^	13.7^A^	7.9^b^	12.6^B^	2.9^c^	12.2^C^	3.9^d^	11.7^D^	4.1^e^	11.3^E^	8.4^f^	10.3^F^
MYC	43.4^a^	56.6^A^	1.4^b^	55.8^B^	0.4^c^	55.6^C^	1.3^d^	54.9^D^	1.3^d^	54.2^E^	74.3^e^	13.9^F^
TEB	50.6^a^	49.4^A^	19.6^b^	39.7^B^	2.9^c^	38.5^C^	6.8^d^	35.9^D^	7.9^e^	33.0^E^	20.5^f^	26.3^F^
OXA	29.1^a^	70.9^A^	57.7^b^	30.0^B^	10.9^c^	26.8^C^	5.1^d^	25.4^D^	0.8^e^	25.2^E^	11.4^f^	22.3^F^


***Cabernet Sauvignon***												
	Destemmed grapes		Pressed must		Fermentation		Racked wines		Clarified wines		Filtered wines	
	Red (%)	Transf (%)	Red (%)	Transf (%)	Red (%)	Transf (%)	Red (%)	Transf (%)	Red (%)	Transf (%)	Red (%)	Transf (%)
ACE	30.1^a^	69.9^A^	5.5^b^	66.1^B^	7.2^c^	61.3^C^	6.3^d^	57.5^D^	4.8^e^	54.7^E^	10.8^f^	48.8^F^
CHL	3.8^a^	96.2^A^	4.4^b^	91.9^B^	15.1^c^	78.1^C^	4.9^d^	74.2^D^	4.5^e^	70.9^E^	14.1^f^	60.9^F^
DEL	25.8^a^	74.2^A^	4.4^b^	70.9^B^	3.4^c^	68.5^C^	4.2^d^	65.7^D^	13.8^e^	56.6^E^	57.1^f^	24.3^F^
IPR	16.5^a^	83.5^A^	3.3^b^	80.8^B^	2.6^c^	78.6^C^	12.3^d^	69.0^D^	2.8^e^	67.1^E^	35.5^f^	43.3^F^
MYC	4.2^a^	95.8^A^	12.4^b^	83.9^B^	6.3^c^	78.7^C^	2.3^d^	76.9^D^	9.3^e^	69.7^E^	10.2^f^	62.6^F^
TEB	12.3^a^	87.7^A^	6.5^b^	81.9^B^	6.7^c^	76.4^C^	6.3^d^	71.7^D^	15.4^e^	60.6^E^	1.5^f^	59.7^F^
OXA	48.8^a^	51.2^A^	16.6^b^	42.7^B^	7.2^c^	39.6^C^	2.4^d^	38.7^D^	4.2^e^	37.0^E^	18.8^f^	30.1^F^

Values represent means of three replicates (*n* = 3). Red (%) = reduction; Transf (%) = transfer rate. For each compound, different superscript letters within reduction (a–f) and transfer rate (A–F) columns indicate significant differences between winemaking stages (ANOVA, followed by Tukey's HSD test, p < 0.05).

ACE – Acetamiprid, CHL – Chlorantraniliprole, DEL – Deltamethrin, IPR – Iprovalicarb, MYC – Mycobutanil, TEB – Tebuconazole, OXA – Oxathiapiprolin.

For *Fetească neagră*, the TR from fresh to destemmed grapes ranged from 13.68–92.65% (iprovalicarb to deltamethrin), fresh grapes to pressed must 1.42–57.66% (myclobutanil to oxathiapiprolin), and fresh grapes to final wine 10.30–82.31% (iprovalicarb - to deltamethrin). Corresponding PFs ranged 0.14–1.00 overall. For *Cabernet Sauvignon*, TR from fresh to destemmed grapes was 51.19–96.17% (oxathiapiprolin to chlorantraniliprole) and fresh grapes to wine 24.25–78.68% (deltamethrin to myclobutanil) with PFs 0.51–0.99. Varietal differences, such as myclobutanil's higher TR in Cabernet Sauvignon, resulted from skin-to-must ratio variations and cultivar-specific maceration dynamics affecting alcohol-soluble compounds extraction.

TR and PF strongly correlated with physicochemical properties. Water solubility negatively correlated with TR from fresh to destemmed grapes (e.g., low-solubility oxathiapiprolin: high TR; higher-solubility myclobutanil: lower TR in *Cabernet Sauvignon)*.TR to pressed must positively correlated with the log *P* value, while TR to wine negative correlated, driven by hydrophobic compounds like deltamethrin (log *P* = 6.2) partitioning into skins /pomace or adsorbing to yeast lees during alcoholic fermentation (41–82%). These significant variations during fermentation suggest a secondary mitigation mechanism such as biosorption onto the yeast biomass. The yeast cell walls act as a adsorbent for hydrophobic residues, which are subsequently removed during racking, effectively reducing the residue profile at these stage.

PF values below 1.00 across all stages indicate significant residue mitigation, with relative stability between the pressed must and clarified wine stages suggestion that, once fermentation is complete, remaining residues reach a physicochemical equilibrium within the wine colloidal matrix and become more resistant to further removal. Higher – TR compounds (chlorantraniliprole, myclobutanil, deltamethrin, tebuconazole) exhibited elevated PFs, while lower – TR (oxathiapiprolin and acetamiprid) showed reduced PFs. Initial residue levels (1.82–18.90 ng/g in *Fetească neagră* and 0.86–8.24 ng/g in *Cabernet Sauvignon*) dropped substantially (PFs 0.14–0.99 and 0.51–0.99, respectively), aligning with safety standards for consumer health (Rahimi et al., 2022; Shabeer et al., 2023).

Our findings align with previous studies by Hou et al. (2020), Kittelmann et al. (2025) and Corrias et al. (2021), confirming that pesticides with higher lipophilicity tend to accumulate in pomace rather than transferring onto the final wine. The relative stability of PF values between pressed must and clarified wine underscore that winemaking effectively reduces pesticide residues, while certain lipophilic compounds may persist to the final product. This relative stability suggests that once fermentation is complete, remaining residues reach a physicochemical equilibrium within the wine colloidal matrix, becoming more resistant to further removal until physical intervention occurs.

### 3.3 Fate and reduction of pesticide residues throughout the winemaking process

In this study, seven pesticides (acetamiprid, chlorantraniliprole, deltamethrin, iprovalicarb, myclobutanil, tebuconazole, and oxathiapiprolin) were quantified across all analyzed matrices, from fresh grapes to filtered wines, using the methodology previously validated by Dumitriu Gabur et al. (2022).

Following destemming and pressing, residue levels of all pesticides decreased markedly, particularly for iprovalicarb (86.32%) and acetamiprid (53.14%) in *Fetească neagră* (Table 3). In contrast, *Cabernet Sauvignon* showed more moderate reductions, with oxathiapiprolin decreasing by 48.80% and acetamiprid by 30.06%. These findings confirm that the greatest decrease in residues occurs during the early winemaking stages, mainly due to the removal of pesticide-laden solid fractions (skins and pomace). Consistent with these observations, Gonzalez-Rodrıguez et al. (2009) reported that approximately half of iprovalicarb residues were removed through absorption into the must, highlighting that the extent of pesticide reduction can vary with grape variety and processing conditions.

In subsequent stages, certain pesticide residues remained relatively stable in both *Fetească neagră* and *Cabernet Sauvignon*, although chlorantraniliprole, deltamethrin, and tebuconazole showed more pronounced decreases during intermediate steps. Further reductions were observed during clarification and filtration, attributed to the adsorption of residues by clarifying agents and filtration media. The filtration step, as the final winemaking stage, played a key role in further reducing pesticide levels. Final concentrations of pesticides in filtered *Fetească neagră* wines were 1.36 ng/g (acetamiprid), 7.86 ng/g (chlorantraniliprole), 0.75 ng/g (deltamethrin), 0.58 ng/g (iprovalicarb), 0.32 ng/g (myclobutanil), 1.12 ng/g (tebuconazole), and 1.44 ng/g (oxathiapiprolin). Corresponding concentrations in *Cabernet Sauvignon* were 0.58 ng/g, 5.02 ng/g, 0.47 ng/g, 0.37 ng/g, 1.04 ng/g, 1.29 ng/g, and 1.08 ng/g, respectively (Fig. 2). These results are comparable to findings by Pinheiro et al. (2025), although our study achieved even lower final concentrations than the 11.17–34.27 μg/L range reported for South American wines.

The clarification stage was crucial in reducing residues through adsorption onto solid deposits. The use of bentonite, a natural montmorillonite clay with a large surface area, significantly contributed to stabilization by binding pesticide residues. While racking caused moderate reductions, the entire winemaking process facilitated an overall reduction of around 90% in total pesticide residues.

Mitigating these residues is critical not only for consumer safety but also for preserving the integrity of natural fermentation. Triazole fungicides, such as tebuconazole, have been shown to inhibit *Saccharomyces cerevisiae* growth even at MRL levels, altering essential metabolic pathways including alanine, aspartate, and glutamate metabolism (Song et al., 2022). Furthermore, Zhao et al. (2023) noted that tebuconazole can disrupt yeast metabolism to the extent of reducing floral volatiles and inducing a brick-red color shift due to increased pyranoanthocyanin formation.

During winemaking, grape-borne pesticides migrate into the must and can influence yeast selection and metabolism. Yeast activity, along with adsorption onto the lees, contributes significantly to residue reduction, while pressing concentrates pesticides in skins and pulp. Technological interventions, including fermentation, racking, clarification, and filtration, further decrease pesticide levels, as observed for pyrimethanil and pyraclostrobin (0.15–0.94 mg/kg, below China's MRL of 2.0 mg/kg), for acetamiprid in grapes (108 μg/kg, Montiel-León et al., 2019), and other seven pesticides (Cermeño et al., 2025).

Our results highlight that the fate of residues is determined by a dominant role of physical removal mechanisms and the inherent physicochemical properties of the pesticides. The strong correlations observed between log *P*, water solubility, and residue activity suggest that these parameters are reliable predictors for the fate of xenobiotics in oenology, providing a robust framework for predicting residue behavior in diverse viticultural scenarios.

### 3.4 Estimated daily intake of pesticide residues from wine consumption and health risk assessment

This study evaluated the Estimated Daily Intake (EDI) and Hazard Quotients (HQ) for seven pesticides throughout the winemaking process of *Fetească neagră* and *Cabernet Sauvignon*, accounting for gender-specific exposure (Table 4, Table 5).

**Table 4:** The estimated daily intake and hazard quotient in the *Fetească neagră* wines.

***Fetească neagră***														
	Fresh grapes		Destemmed grapes		Pressed must		Fermentation		Racked wines		Clarified wines		Filtered wines	
	Women	Men	Women	Men	Women	Men	Women	Men	Women	Men	Women	Men	Women	Men
**Estimated Daily Intake (EDI) (mg/kg bw/day)**														

ACE	1.37E-06	1.16E-06	4.19E-06	7.10E-06	3.65E-06	6.19E-06	3.58E-06	6.07E-06	3.30E-06	5.60E-06	3.07E-06	5.21E-06	2.84E-06	4.81E-06
CHL	6.04E-06	5.12E-06	2.61E-05	4.42E-05	2.11E-05	3.58E-05	1.95E-05	3.30E-05	1.92E-05	3.25E-05	1.68E-05	2.85E-05	1.64E-05	2.77E-05
DEL	5.81E-07	4.92E-07	3.51E-06	5.94E-06	3.30E-06	5.59E-06	3.12E-06	5.28E-06	2.80E-06	4.74E-06	2.15E-06	3.64E-06	1.56E-06	2.64E-06
IPR	1.79E-06	1.51E-06	1.59E-06	2.70E-06	1.47E-06	2.48E-06	1.42E-06	2.41E-06	1.37E-06	2.32E-06	1.31E-06	2.22E-06	1.20E-06	2.03E-06
MYC	7.42E-07	6.29E-07	2.74E-06	4.65E-06	2.70E-06	4.58E-06	2.69E-06	4.56E-06	2.66E-06	4.50E-06	2.62E-06	4.45E-06	6.76E-07	1.14E-06
TEB	1.36E-06	1.15E-06	4.39E-06	7.43E-06	3.53E-06	5.98E-06	3.42E-06	5.80E-06	3.19E-06	5.40E-06	2.94E-06	4.98E-06	2.33E-06	3.96E-06
OXA	2.07E-06	1.75E-06	9.57E-06	1.62E-05	4.05E-06	6.86E-06	3.61E-06	6.12E-06	3.43E-06	5.80E-06	3.40E-06	5.76E-06	3.01E-06	5.10E-06
							

**Hazard Quotient (HQ)**														

ACE	5.48E-05	4.64E-05	1.68E-04	2.84E-04	1.46E-04	2.48E-04	1.43E-04	2.43E-04	1.32E-04	2.24E-04	1.23E-04	2.08E-04	1.14E-04	1.93E-04
CHL	3.87E-06	3.28E-06	1.67E-05	2.84E-05	1.35E-05	2.30E-05	1.25E-05	2.12E-05	1.23E-05	2.08E-05	1.08E-05	1.83E-05	1.05E-05	1.78E-05
DEL	5.81E-05	4.92E-05	3.51E-04	5.94E-04	3.30E-04	5.59E-04	3.12E-04	5.28E-04	2.80E-04	4.74E-04	2.15E-04	3.64E-04	1.56E-04	2.64E-04
IPR	1.19E-04	1.01E-04	1.06E-04	1.80E-04	9.78E-05	1.66E-04	9.49E-05	1.61E-04	9.12E-05	1.54E-04	8.74E-05	1.48E-04	8.00E-05	1.36E-04
MYC	2.97E-05	2.52E-05	1.10E-04	1.86E-04	1.08E-04	1.83E-04	1.08E-04	1.82E-04	1.06E-04	1.80E-04	1.05E-04	1.78E-04	2.70E-05	4.58E-05
TEB	4.54E-05	3.85E-05	1.46E-04	2.48E-04	1.18E-04	1.99E-04	1.14E-04	1.93E-04	1.06E-04	1.80E-04	9.79E-05	1.66E-04	7.78E-05	1.32E-04
OXA	1.48E-05	1.25E-05	6.84E-05	1.16E-04	2.89E-05	4.90E-05	2.58E-05	4.37E-05	2.45E-05	4.15E-05	2.43E-05	4.11E-05	2.15E-05	3.64E-05
(HI)	3.26E-04	2.76E-04	9.66E-04	1.64E-03	8.42E-04	1.43E-03	8.10E-04	1.37E-03	7.52E-04	1.27E-03	6.63E-04	1.12E-03	4.86E-04	8.24E-04

ACE – Acetamiprid, CHL – Chlorantraniliprole, DEL – Deltamethrin, IPR – Iprovalicarb, MYC – Myclobutanil, TEB – Tebuconazole, OXA – Oxathiapiprolin.

**Table 5:** The estimated daily intake and hazard quotient in the *Cabernet Sauvignon* wines.

*Cabernet Sauvignon*														
	Fresh grapes		Destemmed grapes		Pressed must		Fermentation		Racked wines		Clarified wines		Filtered wines	
	Women	Men	Women	Men	Women	Men	Women	Men	Women	Men	Women	Men	Women	Men
	**Estimated Daily Intake (EDI) (mg/kg bw/day)**													
ACE	3.79E-07	3.21E-07	1.73E-06	2.93E-06	1.63E-06	2.77E-06	1.51E-06	2.57E-06	1.42E-06	2.40E-06	1.35E-06	2.29E-06	1.21E-06	2.04E-06
CHL	2.63E-06	2.23E-06	1.65E-05	2.80E-05	1.58E-05	2.67E-05	1.34E-05	2.27E-05	1.27E-05	2.16E-05	1.22E-05	2.06E-05	1.05E-05	1.77E-05
DEL	6.23E-07	5.28E-07	3.01E-06	5.10E-06	2.88E-06	4.88E-06	2.78E-06	4.72E-06	2.67E-06	4.52E-06	2.30E-06	3.89E-06	9.85E-07	1.67E-06
IPR	2.74E-07	2.32E-07	1.49E-06	2.53E-06	1.45E-06	2.45E-06	1.41E-06	2.38E-06	1.23E-06	2.09E-06	1.20E-06	2.03E-06	7.75E-07	1.31E-06
MYC	5.32E-07	4.51E-07	3.33E-06	5.64E-06	2.91E-06	4.94E-06	2.73E-06	4.63E-06	2.67E-06	4.52E-06	2.42E-06	4.10E-06	2.17E-06	3.68E-06
TEB	6.92E-07	5.86E-07	3.95E-06	6.70E-06	3.70E-06	6.26E-06	3.45E-06	5.84E-06	3.23E-06	5.48E-06	2.73E-06	4.63E-06	2.69E-06	4.57E-06
OXA	1.15E-06	9.74E-07	3.84E-06	6.50E-06	3.20E-06	5.42E-06	2.97E-06	5.03E-06	2.90E-06	4.91E-06	2.78E-06	4.70E-06	2.25E-06	3.82E-06

**Hazard Quotient (HQ)**														
ACE	1.51E-05	1.28E-05	6.91E-05	1.17E-04	6.53E-05	1.11E-04	6.06E-05	1.03E-04	5.68E-05	9.62E-05	5.40E-05	9.16E-05	4.82E-05	8.17E-05
CHL	1.69E-06	1.43E-06	1.06E-05	1.79E-05	1.01E-05	1.71E-05	8.59E-06	1.46E-05	8.17E-06	1.38E-05	7.80E-06	1.32E-05	6.70E-06	1.14E-05
DEL	6.23E-05	5.28E-05	3.01E-04	5.10E-04	2.88E-04	4.88E-04	2.78E-04	4.72E-04	2.67E-04	4.52E-04	2.30E-04	3.89E-04	9.85E-05	1.67E-04
IPR	1.83E-05	1.55E-05	9.97E-05	1.69E-04	9.64E-05	1.63E-04	9.38E-05	1.59E-04	8.23E-05	1.39E-04	8.00E-05	1.36E-04	5.17E-05	8.75E-05
MYC	2.13E-05	1.80E-05	1.33E-04	2.25E-04	1.17E-04	1.97E-04	1.09E-04	1.85E-04	1.07E-04	1.81E-04	9.68E-05	1.64E-04	8.69E-05	1.47E-04
TEB	2.31E-05	1.95E-05	1.32E-04	2.23E-04	1.23E-04	2.09E-04	1.15E-04	1.95E-04	1.08E-04	1.83E-04	9.12E-05	1.54E-04	8.98E-05	1.52E-04
OXA	8.21E-06	6.96E-06	2.74E-05	4.64E-05	2.29E-05	3.87E-05	2.12E-05	3.59E-05	2.07E-05	3.51E-05	1.98E-05	3.36E-05	1.61E-05	2.73E-05
(HI)	1.50E-04	1.27E-04	7.73E-04	1.31E-03	7.23E-04	1.22E-03	6.87E-04	1.16E-03	6.49E-04	1.10E-03	5.80E-04	9.82E-04	3.98E-04	6.74E-04

ACE – Acetamiprid, CHL – Chlorantraniliprole, DEL – Deltamethrin, IPR – Iprovalicarb, MYC – Myclobutanil, TEB – Tebuconazole, OXA – Oxathiapiprolin.

For *Fetească neagră*, EDI values (mg/kg body weight/day) decreased progressively from fresh grapes to filtered wine, indicating a reduction in pesticide residues during winemaking. For example, the EDI of acetamiprid in women declined from 1.37 × 10^−6^ in fresh grapes to 2.84 × 10^−7^ in filtered wine. Similar decreasing trends were observed for all analyzed pesticides, with only minor gender-related differences. Correspondingly, HQ values remained well below the safety threshold (HQ < 1) and decreased at each processing step. The cumulative hazard index (HI) declined from 3.26 × 10^−4^ (women) and 2.76 × 10^−4^ (men) in fresh grapes to 4.86 × 10^−5^ and 8.24 × 10^−5^, respectively, in filtered wine, confirming a substantial reduction in overall exposure risk (Table 4).

Comparable results were obtained for *Cabernet Sauvignon*. EDI and HQ values for all pesticides decreased across successive stages, reflecting a consistent decline in residue concentrations. Deltamethrin exhibited the highest HQ values, 3.01 × 10^−4^ for women and 5.10 × 10^−4^ for men, at the destemming stage; however, these values remained far below the acceptable threshold. The HI values followed the same pattern, decreasing from 1.50 × 10^−4^ (women) and 1.27 × 10^−4^ (men) in fresh grapes to 3.98 × 10^−5^ and 6.74 × 10^−5^, respectively, in filtered wine (Table 5).

Overall, both wine varieties showed a consistent decrease in EDI, HQ, and HI values throughout the winemaking process. These results indicate that winemaking stages, such as destemming, pressing, fermentation, clarification, and filtration, effectively reduce pesticide residues and potential dietary exposure. The low HQ and HI values observed in filtered wines suggest minimal health risks associated with the consumption of *Fetească neagră* and *Cabernet Sauvignon* wines. Vertuccio et al. (2025) found that all HQ values (4.49 × 10^−6^ to 5.50 × 10^−5^) were below 1, suggesting that the detected residues in grapes posed no health risks and were safe for human consumption.

The health risk assessment further aimed to evaluate the potential non-carcinogenic effects of pesticides introduced to humans through wine consumption. The risks associated with pesticide exposure were quantified using the Hazard Quotient (HQ), a widely recognized indicator of chemical safety, calculated as the ratio between the estimated daily intake of a pesticide and its established reference dose. The HQ values for all analyzed pesticides were below the threshold of 1 for red wines, indicating that a daily consumption of 150 mL does not pose a significant health risk to consumers. Similarly, the Hazard Index (HI), representing the cumulative risk from multiple pesticide exposures, also remained below 1 for all wine types (Table 4, Table 5). These findings confirm that, under typical consumption scenarios, pesticide residues in wine are unlikely to represent a substantial health concern.

Given the observed low risk levels, preventive and remediation strategies remain essential to maintain this safety margin throughout production. Preventive approaches include the use of biological control methods, biodynamic or integrated vineyard management, limiting post-harvest grape storage, and initiating fermentation immediately after destemming. When contamination is detected, remediation strategies may involve the application of selected yeast strains capable of adsorbing pesticide residues during fermentation, the use of uncontaminated pomace as an adsorbent, and the incorporation of fining agents to further reduce residue levels.

Among the studied pesticides, tebuconazole, a triazole fungicide widely used in viticulture to control downy and powdery mildew (Gur et al., 2022) was frequently detected in red wine (Pérez-Mayán et al., 2020). Chronic exposure to tebuconazole has been associated with hepatotoxicity through mitochondrial dysfunction, genotoxic effects on antibiotic resistance genes, reproductive toxicity, and apoptosis induction (Gao et al., 2024; Leite et al., 2024). Moreover, tebuconazole can influence the organoleptic characteristics of wines, including taste and color, particularly in *Cabernet Sauvignon* and *Merlot* varieties (Zhao et al., 2022).

According to Fernandes Pinheiro et al. (2025), while numerous pesticides may be applied in the vineyard, many are degraded during maturation (up to 89.5% reduction) or eliminated through the chemical and enzymatic reactions inherent to winemaking. Moreover, the actual risk is further modulated by pesticide bioaccessibility, as noted by Liu et al. (2023), bacterial activity in the colon plays a critical role in the final degradation and absorption dynamics within the human body.

While our study found low HQ and HI values, confirming that winemaking acts as an effective barrier to pesticide transfer, the toxicological profiles of other detected classes cannot be ignored. For instance, chronic exposure to chlorantraniliprole has been reported to adversely affect intestinal and neural health through oxidative imbalance and metabolic disturbances (Wang et al., 2025). Similarly, neonicotinoids like acetamiprid are associated with neurotoxic effects and potential reproductive dysfunction upon chronic contact (Han et al., 2018; Phogat et al., 2022).

In conclusion, these findings highlight the dominant role of both viticultural and oenological practices in determining the final residue load. Ongoing surveillance across multiple pesticide classes remains imperative to protect consumer health and ensure the premium quality of wine products.

### 3.5 Cluster analysis

**Fig. 3:**
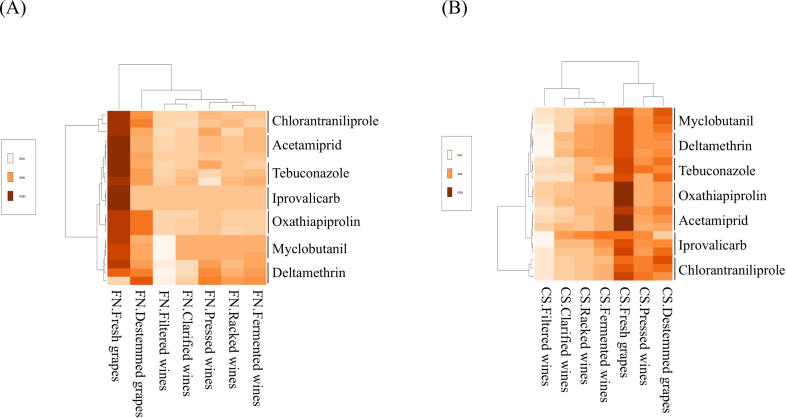
Heatmap analysis of pesticide residue concentrations across the winemaking stages of *Fetească neagră* (A) and *Cabernet Sauvignon* (B)

The heatmaps illustrate the evolution of pesticide residues throughout the winemaking process for *Cabernet Sauvignon* (CS) and *Fetească neagră* (FN). In both varieties, the initial stages (fresh and destemmed grapes) exhibited the highest concentrations of most residues, particularly oxathiapiprolin, iprovalicarb, and chlorantraniliprole, suggesting limited degradation or removal before fermentation. However, a clear downward trend was observed as processing advanced, with the lowest levels detected in clarified and filtered wines, indicating effective dissipation or adsorption of residues during winemaking (Fig. 3A and B).

Notably, *Cabernet Sauvignon* samples showed slightly higher overall residue intensities compared to *Fetească neagră*, especially for oxathiapiprolin and tebuconazole, which appeared more persistent in the *Cabernet Sauvignon* matrix. This may be related to varietal differences in grape skin thickness, wax composition, and must composition, all of which can influence pesticide adsorption and solubility. In contrast, *Fetească neagră* demonstrated a more uniform and rapid decrease of residues after fermentation, suggesting more efficient transfer reduction mechanisms during processing.

Clustering analysis further supports this behavior, clearly separating pre-fermentative and post-fermentative stages in both varieties. Fermentation, clarification, and filtration emerged as key technological steps responsible for significant residue removal. Overall, these results confirm that most pesticide residues are substantially reduced during winemaking, with minimal amounts remaining in the final wines, consistent with regulatory safety limits and previous findings for red wine production.

The heatmap generated from the hierarchical cluster analysis clearly illustrates the dynamics of pesticide residue concentrations across the winemaking stages of *Fetească neagră* (Fig. 3A). A distinct gradient is observed, with fresh and destemmed grape samples exhibiting the highest pesticide levels, as indicated by the darker shades of brown. This confirms that the initial stages of winemaking carry the greatest pesticide burden. As the process advances through pressing, fermentation, racking, clarification, and filtration, a progressive and substantial reduction in pesticide concentrations occurs, reflected by the transition to lighter colors on the heatmap. Certain pesticides such as chlorantraniliprole and acetamiprid show relatively higher persistence during intermediate stages, whereas others, like deltamethrin and oxathiapiprolin, appear to degrade more rapidly.

For *Fetească neagră* variety, two distinct clusters were identified: one comprising the fresh grape samples, and the other including all subsequent winemaking stages. For *Cabernet Sauvignon* (Fig. 3B), the dendrogram revealed two distinct clusters: the first comprising fresh grape, destemmed grape, and pressed wine samples, and the second including the subsequent stages of fermentation, racking, clarification, and filtration.

These findings highlight notable differences between the winemaking stages, suggesting that the technological steps exert a significant influence on the persistence and concentration of pesticide residues. These results confirm substantial reductions during the winemaking process, aligning with regulatory limits and prior studies.

## 4 Conclusions

The residue concentration of all seven analyzed pesticides decreased progressively from grapes to the final wine samples, ensuring a substantial reduction in the overall chemical load. The broader dissipation range observed *in Fetească neagră* suggests that varietal-specific matrix effects may enhance the removal of active compounds more effectively than in *Cabernet Sauvignon*. Notably, the highest removal efficiencies were recorded for iprovalicarb (89.70%) and myclobutanil (86.05%) in *Fetească neagră,* while deltamethrin exhibited its peak reduction (75.74%) in the *Cabernet Sauvignon* variety.

Physical processes such as pressing, fermentation, clarification, and filtration drive these reductions, with the efficiency of removal influenced by pesticide physicochemical properties; more lipophilic compounds tend to accumulate in grape solids, while more water-soluble residues are more effectively eliminated. Heatmap and clustering analyses confirmed the progressive decline of residues and highlighted varietal differences in compound persistence.

Correspondingly, EDI, HQ, and HI values decreased throughout winemaking, remaining well below safety thresholds, indicating minimal health risks for consumers under typical consumption scenarios.

These findings underscore the critical role of both viticultural management and oenological processes in mitigating pesticide exposure and safeguarding wine quality. While the winemaking process acts as a natural purification system, preventive and remediation strategies, such as integrated vineyard management and yeast-mediated adsorption can further enhance this safety margin. Building upon these strategies, our future research will focus on the principles of the circular economy by evaluating the use of grape pomace as a sustainable, bio-based fining agent for pesticide residue removal. This innovative approach aims to repurpose viticultural by-products into high-value tools, ensuring that final wines remain safe, high-quality, and strictly compliant with evolving consumer health standards.

## CRediT authorship contribution statement

**Georgiana-Diana Gabur:** Writing – original draft, Project administration, Methodology, Investigation, Data curation, Conceptualization. **Valeriu V. Cotea:** Supervision, Conceptualization. **Daniela Fighir:** Writing – review & editing, Data curation. **Carmen Teodosiu:** Writing – review & editing, Supervision, Conceptualization. **Iulian Gabur:** Writing – review & editing, Supervision, Data curation.

## Declaration of competing interest

The authors declare that they have no known competing financial interests or personal relationships that could have appeared to influence the work reported in this paper.

## Data Availability

Data will be made available on request.
