# Supplementary material for: Dissipation behavior and dietary risk assessment of pesticide residues during the winemaking of Fetească neagră and CabernetSauvignon
Source: Food Chem X. 2026 May 10;36:103930. doi: 10.1016/j.fochx.2026.103930 (PMC13202246; doi:10.1016/j.fochx.2026.103930)
Supplement: Supplementary file 1 — Supplementary material [file mmc1.docx]

**Supplementary Material**

**Dissipation behavior and dietary risk assessment of pesticide residues during the winemaking of Fetească neagră and Cabernet Sauvignon**

Table 1. Phytosanitary treatment schedule applied in the vineyard during 2020

| Application | Date | Phytosanitary treatments |
| --- | --- | --- |
| 1 | 20–24 May | Zorvec Zelavin®: Oxathiapiprolin  Systhane Forte: Myclobutanil |
| 2 | 03–06 June | Melody compact: Iprovalicarb  Folicur: Tebuconazol  Coragen: Chlorantraniliprole |
| 3 | 10–14 June | Zorvec Zelavin®: Oxathiapiprolin  Folicur: Tebuconazol |
| 4 | 19–24 June | Coragen: Chlorantraniliprole  Systhane Forte: Myclobutanil  Zorvec Zelavin ®: Oxathiapiprolin |
| 5 | 02-06 July | Decis Expert: Deltamethrin  Bouillie Bordellaisse: Copper |
| 6 | 10–14 July | Systhane Forte: Myclobutanil  Bouillie Bordellaisse: Copper  Decis Expert: Deltamethrin |
| 7 | 28–31 July | Gazelle: Acetamiprid  Bouillie Bordellaisse: Copper |
| 8 | 02–04 August | Gazelle: Acetamiprid  Bouillie Bordellaisse: Copper |

Table 2. LC–MS/MS parameters used for the determination of targeted pesticides in wines

| Pesticides | Retention time (min) | Q1  (*m*/*z*) | Q3  (*m*/*z*) | Calubration curve | R^2^ | LOD  (ng/g) | LOQ  (ng/g) | Mean Calculated Concentration  (ng/mL) | Recovery (%) | Standard Deviation  (ng/mL) | Relative Standard Deviation  (RSD %) | Response Factor |
| --- | --- | --- | --- | --- | --- | --- | --- | --- | --- | --- | --- | --- |
| Acetamiprid | 4.44 | 223.2 | 126.0 | y = 0.933x + −0.192 | 0.994 | 0.12 | 0.40 | 2.46 | 99.5 | 0.08 | 3.2 | 2.93 |
|  |  |  |  |  |  |  |  | 4.98 | 101.0 | 0.40 | 8.0 | 3.41 |
|  |  | Confirmation transition: | |  |  |  |  | 9.92 | 100.5 | 0.19 | 1.9 | 2.96 |
|  |  |  |  |  |  |  |  | 19.35 | 98.2 | 1.13 | 5.9 | 3.37 |
|  |  | 223.200 | 90.0 |  |  |  |  | 29.76 | 100.5 | 1.68 | 5.6 | 3.22 |
|  |  |  |  |  |  |  |  | 49.41 | 100.2 | 1.39 | 2.8 | 3.46 |
| Chlorantraniliprole | 5.26 | 481.9 | 283.9 | y = 0.906x + −0.253 | 0.995 | 0.07 | 0.22 | 2.54 | 100.1 | 0.06 | 2.2 | 0.87 |
|  |  |  |  |  |  |  |  | 5.07 | 100.0 | 0.52 | 10.2 | 1.04 |
|  |  | Confirmation transition: | |  |  |  |  | 10.17 | 100.7 | 0.44 | 4.3 | 1.00 |
|  |  |  |  |  |  |  |  | 19.95 | 98.3 | 0.93 | 4.7 | 1.01 |
|  |  | 481.9 | 450.9 |  |  |  |  | 30.72 | 101.1 | 1.73 | 5.6 | 1.06 |
|  |  |  |  |  |  |  |  | 50.66 | 99.9 | 1.82 | 3.6 | 1.08 |
| Iprovalicarb | 5.07 | 321.2 | 119.1 | y = 0.113x + −0.0535 | 0.994 | 0.04 | 1.62 | 2.58 | 102.8 | 0.17 | 6.6 | 3.54 |
|  |  |  |  |  |  |  |  | 5.04 | 100.2 | 0.44 | 8.6 | 4.12 |
|  |  | Confirmation transition: | |  |  |  |  | 10.00 | 99.1 | 0.33 | 3.3 | 3.52 |
|  |  |  |  |  |  |  |  | 19.57 | 97.4 | 1.35 | 6.9 | 4.04 |
|  |  | 321.2 | 91.1 |  |  |  |  | 29.72 | 98.7 | 1.48 | 5.0 | 3.95 |
|  |  |  |  |  |  |  |  | 51.23 | 101.8 | 1.85 | 3.6 | 4.14 |
| Myclobutanil | 5.41 | 289.1 | 70.1 | y = 0.298x + −0.125 | 0.990 | 0.22 | 0.74 | 2.52 | 100.1 | 0.19 | 7.6 | 0.46 |
|  |  |  |  |  |  |  |  | 5.03 | 100.1 | 0.39 | 7.8 | 0.44 |
|  |  | Confirmation transition: | |  |  |  |  | 10.17 | 100.7 | 0.25 | 2.5 | 0.41 |
|  |  |  |  |  |  |  |  | 19.94 | 99.2 | 0.85 | 4.2 | 0.47 |
|  |  | 289.1 | 125.2 |  |  |  |  | 30.06 | 99.5 | 1.70 | 5.7 | 0.48 |
|  |  |  |  |  |  |  |  | 50.53 | 100.5 | 1.62 | 3.2 | 0.48 |
| Tebuconazole | 5.58 | 308.2 | 70.0 | y = 0.132x + −0.0859 | 0.993 | 0.17 | 0.59 | 2.62 | 105.2 | 0.20 | 7.6 | 0.35 |
|  |  |  |  |  |  |  |  | 4.91 | 98.4 | 0.39 | 7.9 | 0.4 |
|  |  |  |  |  |  |  |  | 9.68 | 97.1 | 0.36 | 3.7 | 0.35 |
|  |  |  |  |  |  |  |  | 19.58 | 98.4 | 0.80 | 4.1 | 0.42 |
|  |  |  |  |  |  |  |  | 29.76 | 99.5 | 1.79 | 6.0 | 0.39 |
|  |  |  |  |  |  |  |  | 50.61 | 101.4 | 1.94 | 3.8 | 0.42 |
| Oxathiapiprolin | 6.67 | 540.0 | 500.0 | y = 0.183x + −0.144 | 0.992 | 0.47 | 1.58 | 2.63 | 103.9 | 0.16 | 6.1 | 0.24 |
|  |  |  |  |  |  |  |  | 4.94 | 97.4 | 0.38 | 7.7 | 0.32 |
|  |  | Confirmation transition: | |  |  |  |  | 10.11 | 100.1 | 0.32 | 3.1 | 0.3 |
|  |  |  |  |  |  |  |  | 19.73 | 97.2 | 1.31 | 6.7 | 0.34 |
|  |  | 308.2 | 125.1 |  |  |  |  | 30.60 | 100.6 | 2.11 | 6.9 | 0.35 |
|  |  |  |  |  |  |  |  | 51.00 | 100.8 | 2.75 | 5.4 | 0.36 |
